# Morphology-based noninvasive early prediction of serial-passage potency enhances the selection of clone-derived high-potency cell bank from mesenchymal stem cells

**DOI:** 10.1186/s41232-022-00214-w

**Published:** 2022-10-02

**Authors:** Takashi Suyama, Yuto Takemoto, Hiromi Miyauchi, Yuko Kato, Yumi Matsuzaki, Ryuji Kato

**Affiliations:** 1 Department of Life Science, Faculty of Medicine, Shimane University, 89-1 Enya-cho, Izumo, Shimane, 693-8501 Japan; 2 PuREC Co. Ltd, 89-1 Enya-cho, Izumo, Shimane 693-8501 Japan; 3 Department of Basic Medicinal Sciences, Graduate School of Pharmaceutical Sciences, Nagoya University, Tokai National Higher Education and Research System, Furocho, Chikusa-ku, Nagoya, Aichi 464-8601 Japan; 4 Institute of Nano-Life-Systems, Institutes of Innovation for Future Society, Nagoya University, Tokai National Higher Education and Research System, Furocho, Chikusa-ku, Nagoya, Aichi 464-8601 Japan

**Keywords:** Morphological analysis, Mesenchymal stem cells, LNGFR, THY-1, Rapidly expanding clone (REC), Cell bank establishment, Serial-passage potency, Prediction model

## Abstract

**Background:**

Rapidly expanding clones (RECs) are one of the single-cell-derived mesenchymal stem cell clones sorted from human bone marrow mononuclear cells (BMMCs), which possess advantageous features. The RECs exhibit long-lasting proliferation potency that allows more than 10 repeated serial passages in vitro, considerably benefiting the manufacturing process of allogenic MSC-based therapeutic products. Although RECs aid the preparation of large-variation clone libraries for a greedy selection of better-quality clones, such a selection is only possible by establishing multiple-candidate cell banks for quality comparisons. Thus, there is a high demand for a novel method that can predict “low-risk and high-potency clones” early and in a feasible manner given the excessive cost and effort required to maintain such an establishment.

**Methods:**

LNGFR and Thy-1 co-positive cells from BMMCs were single-cell-sorted into 96-well plates, and only fast-growing clones that reached confluency in 2 weeks were picked up and passaged as RECs. Fifteen RECs were prepared as passage 3 (P3) cryostock as the primary cell bank. From this cryostock, RECs were passaged until their proliferation limitation; their serial-passage limitation numbers were labeled as serial-passage potencies. At the P1 stage, phase-contrast microscopic images were obtained over 6–90 h to identify time-course changes of 24 morphological descriptors describing cell population information. Machine learning models were constructed using the morphological descriptors for predicting serial-passage potencies. The time window and field-of-view-number effects were evaluated to identify the most efficient image data usage condition for realizing high-performance serial-passage potency models.

**Results:**

Serial-passage test results indicated variations of 7–13-repeated serial-passage potencies within RECs. Such potency values were predicted quantitatively with high performance (RMSE < 1.0) from P1 morphological profiles using a LASSO model. The earliest and minimum effort predictions require 6–30 h with 40 FOVs and 6–90 h with 15 FOVs, respectively.

**Conclusion:**

We successfully developed a noninvasive morphology-based machine learning model to enhance the efficiency of establishing cell banks with single-cell-derived RECs for quantitatively predicting the future serial-passage potencies of clones. Conventional methods that can make noninvasive and quantitative predictions without wasting precious cells in the early stage are lacking; the proposed method will provide a more efficient and robust cell bank establishment process for allogenic therapeutic product manufacturing.

**Supplementary Information:**

The online version contains supplementary material available at 10.1186/s41232-022-00214-w.

## Background

Mesenchymal stem cells (MSCs) are the most widely studied stem cells for cell-based therapeutic applications [1–3]. It is known that a variety of cell types exist in MSCs [4–7] because conventional MSC processing simply collects the adherent cell fraction from a cell suspension mixture [8, 9]. LNGFR (CD271) and THY-1 (CD90) co-positive cells (LT cells) are among the specific sub-population MSCs in the bone marrow, and they exhibit unique characteristics that help advance MSC-based cell therapy: (1) high proliferation potency, (2) multiple differentiation potencies (adipogenic, osteogenic, and chondrogenic differentiation), (3) low expression of senescence marker SA-beta-gal, and (4) uniform and small size, which allows them to avoid being trapped in the lung capillaries after intravenous administration in a mouse model [10]. Relatively rapidly proliferating clones were observed in single-cell sorted LT cells; they were named “rapidly expanding clones (RECs).” RECs are now expected to enhance the clinical trials for the treatment of hypophosphatasia and spinal canal stenosis [11], because their proliferative potency greatly assists in establishing a cell bank with less heterogeneity.

The expectations for the advancement of MSC-based therapeutic products have grown because MSCs are being used in leading translational studies for clinical applications [3, 12–14]. Thus, there is currently a great demand for the development of enabling technologies that can assist MSC manufacturing [15, 16]. The characteristics of RECs can greatly benefit from reducing two major critical risks in the present MSC manufacturing process to ensure efficient and robust cell manufacturing:

1. Difficulty in controlling quality variations among donors: MSCs have considerable donor variations [17–19], and it is practically impossible to examine sufficient variations in patient cells before developing a robust manufacturing process [20–22]. Thus, handling various unknown donor cells is a considerably risky task in MSC manufacturing, and it is the major cause of unexpected errors that can be difficult to solve. Allogenic cell bank establishment is a practical approach to control starting cell quality, which enables efficient MSC manufacturing. The REC can greatly benefit the process of establishing an allogenic cell bank. RECs can be mass produced by cell sorting, compared to difficulties involved in running large-scale donor selection until an ideal cell bank is obtained; therefore, the selection of the REC is more feasible and efficient. Furthermore, the proliferation potency of RECs considerably assists the success rate of establishing a cell bank while maintaining the banked cells in the earlier culture period. Thus, REC-based cell manufacturing enables more feasible process development and stable quality management.
2. Quality decay during cell expansion culture: MSCs lose their proliferation potency after several passages; in addition, other important quality attributes also degrade during expansion [23–31]. However, achieving a certain cell number is an essential quality criterion in the manufacturing of MSC-based therapeutic products. From a therapeutic perspective, the common protocol of MSC-based therapy requires more than a billion cells per treatment to ensure efficacy [32]. From a manufacturing aspect, the cells in the final cell bank must reach a large cell number to thoroughly test the final product with multiple quality criteria because most tests are invasive and the cells are consumed for each test [33]. However, such a cell expansion process is an unpromised trial with a high risk of failure owing to the quality decay probability. As a fact, the failure of the cell bank establishment can only be found “as a fact” after all the work, and it is difficult to avoid such failure beforehand. Within this context, the high and long-lasting proliferation potency of REC, which enables more than 10 repeats of serial passage, can greatly minimize the risk of cell bank establishment failure and realize the maximum culture efficiency for establishing a rich number of stocks in the earlier passages.

There is a dilemma in the establishment of cell banks for RECs despite the advantageous features of RECs that enable both the low-risk and high-efficiency manufacturing of allogenic therapeutic products. RECs can be produced effectively by cell sorting even from a small volume of cell source, and therefore, the staring clone variation can be greater than the conventional donor waiting for the allogenic cell banks. However, it is expensive to expand many RECs until the stage of the final cell bank; furthermore, final quality tests for assuring the established cell bank can incur further cost and effort. The variation in the starting RECs can trigger the expectation of selecting “better RECs” during the establishment of the cell bank; such expectations can increase the number of multiple cell bank establishments, which are cost- and effort-intensive. In practice, RECs are screened from LT cells using their primary growth speed from a single clone in a 96-well plate; all candidates are further expanded to form multiple “candidate cell banks” to select “the better REC cell bank” with higher quality. Such greedy selection for the better-quality product (= final product with lower risk and higher potency) is possible with RECs; however, it can raise the cost of the total process. Therefore, it is necessary to determine such candidate RECs as early as possible to help minimize excessive work. However, it is extremely difficult to test early-stage cells in the cell bank establishment, especially with single-clone-derived RECs, using conventional cell evaluation methods because most of them are invasive and waste precious cell sources.

**Fig. 1:**
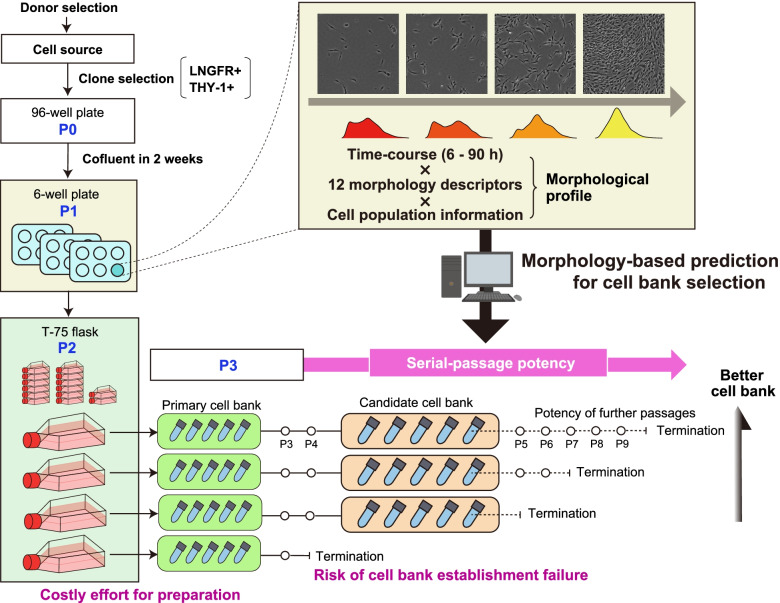
Conceptual illustration of serial-passage potency prediction using a morphological profile for selecting a high-potency cell bank. The target RECs were sorted from the MSCs via the clone selection step (P1 and P2). At P3, cells were cryopreserved to form the primary cell bank for preparing early passage cells for further experiments. Serial-passage tests were examined from P3 till the limitation of the passage. For the practical cell-based therapeutic product manufacturing, the candidate cell bank is formed during such serial passages. However, there are risks; for example, cells show unexpected growth termination, which results in cell bank establishment failure. Furthermore, the formation of a better-quality candidate cell bank which possesses lower risks of having banked cells which loses further proliferation potency but has higher potency of further activity is expected. Such serial-passage potency was predicted from the morphological profile in the P2 stage cell images. The morphological profile comprises time course × 12 morphological descriptors × cell population information (mean and SD)

In this study, we develop a morphology-based noninvasive potency prediction method for selecting “the better RECs” in the early stage of cell bank establishment where the cell number is extremely limited. Based on our previous findings on morphology-based early prediction for MSC quality decay [34–37], we attempt to predict the “further serial-passage potencies” using only their early morphological information to enhance the selection of the better RECs that form the better REC cell bank (Fig. 1). Furthermore, we expect that such potency prediction can help aid the cell bank establishment process to balance the “bank size” and “potency of banked cells” because it is also a critical dilemma for processing the cell bank. Manufacturing efficiency can be increased if a cell bank is largely expanded; however, this increases the risk of losing the proliferation potency of the banked cells. Therefore, we hypothesized that if future serial-passage potencies can be predicted in advance, it will enable to design a low-risk timing to achieve the maximum-sized cell bank which has high potency.

For the training data to develop a prediction model, we established 15 RECs from the bone marrow and experimentally confirmed the serial passage number until passage limitation (defined as “serial-passage potencies”). Using the morphological descriptors in the stage of passage 1 (P1), we attempted to develop machine learning models to quantitatively predict such potency. During the development of prediction models, we conducted a detailed analysis of the data usage effect to obtain high-performance prediction models robustly. Thus, our data show the most effective time-course data usage and the minimum number of images required to realize the prediction model in a practical manner. The future potency prediction concept of our morphology-based REC indicates the potential of an image-based data-driven cell bank construction process in MSC manufacturing that can achieve both efficiency and robustness.

## Methods

### Cells and culture

Bone marrow mononuclear cells were prepared from bone marrow aspirate (AllCells, Alameda, USA) collected from healthy donors using density gradient centrifugation with Ficoll (GE Healthcare, Chicago, USA) to obtain RECs. Bone marrow mononuclear cells were stained with anti-human rabbit anti-CD90 IgG (BD Biosciences, Cat#559,869, Flanklin Lakes, USA) and anti-human mouse anti-CD271 IgG (Thermo Fisher) for 1 h at 37 °C. Single-cell sorting was performed for CD90 and CD271 double-positive cells using the cell sorter (JSAN, BayBioscience, Kobe, Japan). The LT cells were sorted in 96-well plates (Thermo Fisher Scientific, Waltham, USA) as single clones (passage count = P0); they were further cultured with a maintenance medium (low glucose Dulbecco’s modified Eagle’s medium [DMEM] (Wako, Osaka, Japan) containing 20% fetal bovine serum [FBS] (Cytiva HyClone, Marlborough, USA), 20 ng/ml basic fibroblast growth factor [bFGF] (KAKEN PHARMACEUTICAL, Tokyo, Japan), 0.01 M 4-(2-hydroxyethyl)-1-piperazineethanesulfonic acid [HEPES] (Thermo Fisher Scientific, Waltham, USA), and 1% penicillin/streptomycin (Meiji Seika Pharma, Tokyo, Japan)). After 2 weeks, the clones were sub-confluent in 96-well plates, harvested, sub-cultured in a single well of a 6-well plate (Thermo Fisher Scientific) (passage count = P1), and set for image acquisition. Image acquisition for the further morphological analysis is done at this stage. The cells were then harvested and sub-cultured in T75 flasks (Thermo Fisher Scientific) (passage count: P2) when cells reached sub-confluency in a 6-well plate (Thermo Fisher Scientific). The cells were harvested using TrypLE Select (Thermo Fisher Scientific) after incubation for 3–5 min at 37 °C. The P2 cells in the T75 flasks that reached sub-confluent status were cryopreserved with 1.0 × 10^6^ cells/ml with CP-1(KYOKUTO PHARMACEUTICAL, Tokyo, Japan) and 25% human serum albumin (CSL Behring, Tokyo, Japan); they were designated “primary cell bank (P3).” With the primary cell bank, in vitro and in vivo tests for the multiple potencies were excluded to save cells for the serial-passage experiments. For comparing the morphologies, conventionally processed bulk MSCs (BMMSCs: Lot 0,000,394,413, 0,000,411,107, 0,000,413,042, 0,000,422,610, 0,000,423,370, 0,000,429,365, 0,000,446,319, 0,000,451,491, 0,000,458,207) purchased from Lonza Japan, Ltd. (Tokyo, Japan) were cultured to P3 in MSCGM (Lonza Japan, Ltd.) supplemented with BulletKit (Lonza Japan, Ltd.). All cell cultures were maintained at 37 °C and 5% CO_2_; the medium was changed once every 3 days.

### Serial-passage potency test

The primary cell bank (P3) vial was thawed and seeded in a 100-mm dish (Thermo Fisher Scientific) with a density of 2.0 × 10^5^ cells/dish, which we named as P3 data. Cells were harvested from a 100-mm dish using a TrypLE Select (Thermo Fisher Scientific) and sub-cultured in the new 100-mm dish with the same seeding density when the cells reached sub-confluent status. This sub-culture process was repeated until the cell growth was arrested. We counted the passage numbers and designated the final passage number as a “serial-passage limitation number” that we defined as the “serial-passage potency” (Fig. 1). At each passage, the harvested total cell number was counted using Cellometer Auto T4 (Nexcelom Bioscience, Lawrence, USA), and the result was recorded as the total number of collected cells.

### Image acquisition and processing

Phase-contrast microscopic images were acquired every 6 h for P1 RECs in a 6-well plate (Thermo Fisher Scientific) using the automatic image acquisition system BioStation CT (Nikon Corporation, Tokyo, Japan) at × 4 magnification (64 tiling per well, covering 16 mm^2^, 1000 pixels/image). Each clone lot was imaged in one well; the cell number was extremely limited at this stage. For bulk MSCs, the MSCs were seeded into 6-well plates (Corning Incorporated, NY, USA) at 2000 cells/cm^2^ (*n* = 3 wells per lot). The time points were designated as time 1 (6 h after seeding) to 20 (120 h after seeding). Time 15 was selected as the final time point for morphology and growth rate measurements because some lots reached more than the sub-confluent status; therefore, it was difficult to recognize individual cells in the image accurately. Raw images were processed to measure individual cell morphologies for obtaining a summary of morphological profiles of the cell population (minimum of 300 cells to a maximum of 100,000 cells collected per well covered by 64 images). Image processing was performed by original codes using Python version 3.7.3, with packages NumPy version 1.20.0 and OpenCV version 4.4.0. The image processing pipeline was designed with eight processes: (1) background adjustment, (2) enhancement of texture, (3) binarization, (4) removal of small objects, (5) erosion, (6) removal of small objects, (7) fill hole, and (8) removal of frame-touching objects (Supplementary Fig. 1). After image processing, 12 basic morphological descriptors (Supplementary Table 1) were measured per cell region in each image. The summary of cell population was described by calculating “mean” and “standard deviation (SD)” using all single-cell-based morphological descriptor data. This summary represented nearly 1 × 10^4^ to 1 × 10^5^ cells from a single well covered by 64 images. We accumulated this morphological descriptor summary throughout the time course (6–90 h) and designated them as the “morphological profile” of the sample. The complete morphological profile for each condition (= 1 well) comprised 360 parameters (= 12 descriptors × (mean and SD) × 15 time points). All data processing was conducted using the R version 4.0.2.

### Visualization of morphological profiles

Gaussian kernel density estimation was calculated using single-cell data at each time point to visualize the cell population using a single morphological descriptor. The distribution was estimated using a kernel function in R. Raw data were transformed with log10 for distribution estimation to describe the “area.” Single-cell measurement data that exceed area > 200 pixels were collected to allow a detailed “area” discussion between RECs and conventionally processed bulk MSCs. Most objects smaller than 200 pixels were found in “round cells” during their proliferation, and therefore, it was considered difficult to discuss the size difference because the characteristics of expanding cells diminish in such data. The morphological profiles of all lots were analyzed using principal component analysis (PCA) to visualize the relative similarities of clones using multiple descriptors. Dots were colored with clone labels (15 colors) and serial-passage limitation numbers (gradations of seven levels: 7 to 13) in the PCA that compares clone morphological profiles. All data with the same time-window size, including different FOV number usages, were merged and used to set the total principal components for covering total data diversity to visualize the data usage effect and change their time-window size and numbers of FOVs. Then, data using different FOV numbers were plotted individually in the fixed PCA axis. In the comparative PCAs for the data-usage effect, one plot indicates “one clone.” The total single-cell measurement data from each data size (varying the combination of different time-window size and FOV numbers) were resampled by bootstrap (50 repeats allowing overlaps) to visualize the explanatory power of different data usages; their new mean and SD were re-obtained from each resampled data. A total of 50 plots per clone were plotted using PCA and such resampled data. Student’s *t-*test was used to test differences between the morphological descriptors and the population distributions of morphological descriptors. All data processing was performed using R (version 4.0.2).

### Construction of prediction models for serial-passaging potency

Time-course morphological profiles were used as explanatory parameters, and an experimentally determined serial-passage limitation number was used as the objective parameter in the dataset for machine learning. Two types of machine learning models were examined—the linear regression model least absolute shrinkage and selection operator (LASSO) and the nonlinear machine learning model random forest (RF). With LASSO, parameter selection was performed using a mean decreasing Gini index. Model performances were validated by leave-one-out cross-validations and compared by root-mean-squared error (RMSE). The data usage effect in the morphological information was examined with an exhaustive combination of two parameters: length of time window (ranging from 6–90 h) and number of FOVs (ranging from 1 to 64). The total 6–90 h window was shortened from the last time point (90 h) at each time point (6 h) to vary the length of the time window. Thus, the maximum total morphological descriptor comprises 360 parameters, whereas the minimum comprises 24 parameters. Images from 64 images were selected randomly to vary the number of FOVs. The bootstrapping of 50 repeated times of re-sampling FOVs from 64 images was introduced to increase the dataset size from 15 to 750 samples for evaluating the data variation effect on selected data size conditions (6 h, 6–30 h, 6–60 h vs. 15, 40, and 60 FOVs). All data processing and machine learning were performed using the R version 4.0.2.

## Results

### Collection and characterization of RECs for the training data

We started our work to achieve 15 clones of RECs from a single donor of bone marrow mononuclear cells (BMMCs) to develop a machine learning model for predicting “serial-passage potency” from the early-stage cell morphologies (Scheme in Fig. 1). Fifteen clones were collected from the same donor sample and sorted according to the basic criteria for REC: (1) LNGFR and THY-1 double-positive in cell sorting and (2) reaching sub-confluent status within 2 weeks after single-clone sorting in 96-well plates (P0 stage). Then, the clones expanded in a 6-well plate followed by a T-75 flask were cryopreserved as primary cell banks (P3), which is the bank for storing early passage cells.

**Fig. 2:**
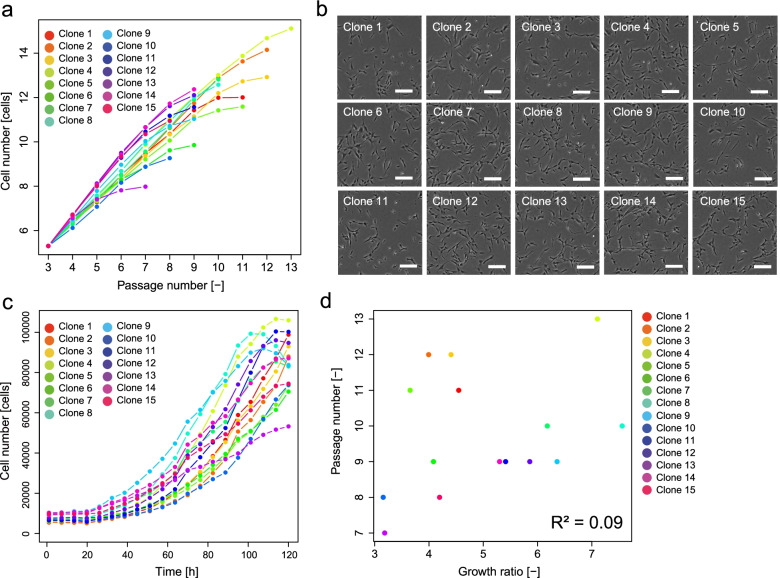
Profile of 15 RECs. **a** Results of serial-passage tests of RECs. **b** Representative morphologies of RECs. **c** Growth profile of RECs. **d** Correlation of growth rate and serial-passage limitation number. *R*^2^ indicates the coefficient of determination

We evaluated the serial-passage potencies of candidate clones in P2 to further select “the better RECs.” The cellular performance that enables several repeated rounds of passages can be considered an ideal criterion for selecting “a better-quality cell bank” for further usage. The serial-passage potency test results indicated that our RECs retained high proliferation potencies, which marks 9.8 repeats of serial passage on average (Fig. 2a). Such a potency can be considered high compared to that of commercially available MSCs processed using conventional methods. However, some clones lost their proliferation gradually, even among clones that passed the REC criteria and retained long serial-passage potencies (e.g., clone 11 or 13 in our data).

Next, we evaluated the morphological characteristics of the clones (Fig. 2b). At the P1 stage in 6-well plates, which is an extremely early stage, the morphology includes an important signature of the cells for their evaluation. However, manual morphological observation (without quantification) makes it difficult to discriminate the differences between the RECs.

We analyzed the growth profiles of RECs at the P1 stage using time-course images (Fig. 2c). Among the 15 clones, 12 showed a growth rate over fourfold; this growth rate was significantly higher than that of several conventionally processed bulk MSCs in our study (Supplementary Fig. 2). The clone with the lowest serial-passage potency (clone 13) showed a low growth rate; the highest serial-passage potency clone (clone 4) showed a high growth rate. However, the coefficient of determination between the “growth rate” and the “serial-passage limitation number” was low (*R*^2^ = 0.09) (Fig. 2d). Thus, the data indicate that the growth rate measurement at the P1 stage cannot predict future serial-passage potencies.

### Morphological characterization of RECs

**Fig. 3:**
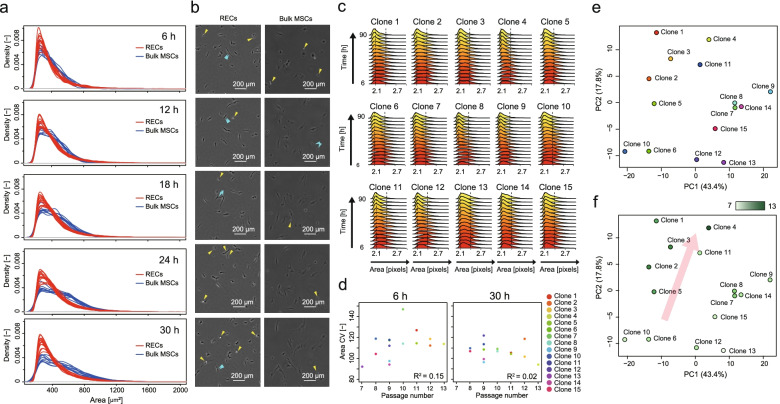
Morphological characterization of 15 RECs. **a** Size distribution comparison between RECs (15 clones) and conventionally processed bulk MSCs (cMSCs, 9 lots) and their time-course changes. Only adherent and extending cells are counted. The dotted vertical line represents the average cell sizes. **b** Representative time-course images of REC and Bulk MSC. Yellow and blue arrows represent proliferating cells and proliferated cells in near time, respectively. **c** Size distribution and their time-course changes among 15 clones. **d** Correlation of “SD of area” and serial-passage limitation number. *R*^2^ indicates the coefficient of determination. **e**, **f** PCA plot of 15 RECs profiled by 24 morphological descriptors. PCA plot with clone color labels (**e**). PCA plot colored by the heatmap of their serial-passage potencies (**f**)

We attempted to characterize RECs (at P1) quantitatively via image-based morphological analysis according to our previously reported analysis concepts [33–36]. Compared with conventionally processed bulk MSCs (cMSCs, nine lots), RECs were more homogeneous and remained smaller even after adhesion (Fig. 3a, b). Both RECs and cMSCs started from a similarly sized population (median area = 358 μm^2^, 335 μm^2^, respectively) at the very early adhesion stage (6 h, *T*-test *p* < 0.22); the median size of the RECs remained small (363 μm^2^) wherein the bulk cMSCs expanded during the culture after 30 h (median area = 473 μm^2^, *T*-test *p* < 0.000001). Furthermore, more proliferating cells that are visually white and round under phase-contrast microscopy were found in the RECs during the same series of time-course images (Fig. 3b).

We found that the adherent cell population remained broad in clones with decreased serial-passage potencies by visualizing the population change of such RECs during the time course at P1 stage (Fig. 3c). Thus, high-potency cells can achieve a homogenous size population; however, such size data analysis was suggestive, and the coefficient of determination between the “SD of area” and the “serial-passage limitation number” remained low (Fig. 3d). A single morphology descriptor analysis was not sufficiently efficient for a quantitative prediction.

We profiled clones using multiple morphological information described with 24 descriptors obtained from the basic descriptors (Supplementary Table 1) [33–36]. The morphological similarities of the RECs are visualized using principal component analysis (PCA) (Fig. 3e is labeled by clone numbers, and Fig. 3f is labeled by the serial-passage-potency of each clone). These results indicate that there are certain clusters of clones with similar morphological profiles that slightly divide the low-and high-serial-passage potency clones. In practice, low serial-passage potency clones gather in the low PC2 axis, whereas the high serial-passage potency clones gather in the high PC2 axis and in the middle of the PC1 axis. The contributing descriptors in the axes of the PCA map, especially in the most explanatory PC2 axis, can be interpreted as follows (Supplementary Table 2): The clone has a longer serial-passage potency when cells are more homogeneous and show a spindle shape during the 24–30-h growth; however, their serial-passage potency is shorter when cellular morphological homogeneity is disturbed. Such an unsupervised model analysis suggests that a multiple-descriptor combination provides a better explanation to morphologically characterize potencies.

### Morphology-based machine learning for predicting serial-passage potency

We next investigated the development of machine learning models with morphological information to enable the quantitative prediction of “serial-passage potencies” of RECs based only on morphological information (Fig. 1). We used morphological profiles (24 descriptors × 15 time points) to predict the serial-passage limitation numbers. Under this prediction model development, we attempted to understand the part or the extent to which the morphological information effectively contributes to the development of the prediction model. Thus, we investigated the effect of morphological data usage by changing two parameters: the time-window effect and the field-of-view (FOV)-number effect. We investigated these parameters because we found that the image data collection effort can be effectively reduced via such detailed investigation in our previous challenge involving the prediction of the growth rate and osteogenic differentiation rate of MSCs from morphological descriptors [33–36]. Shortening the time window and minimizing the FOV number can not only save time and effort for image data acquisition, but can also accelerate the prediction.

**Fig. 4:**
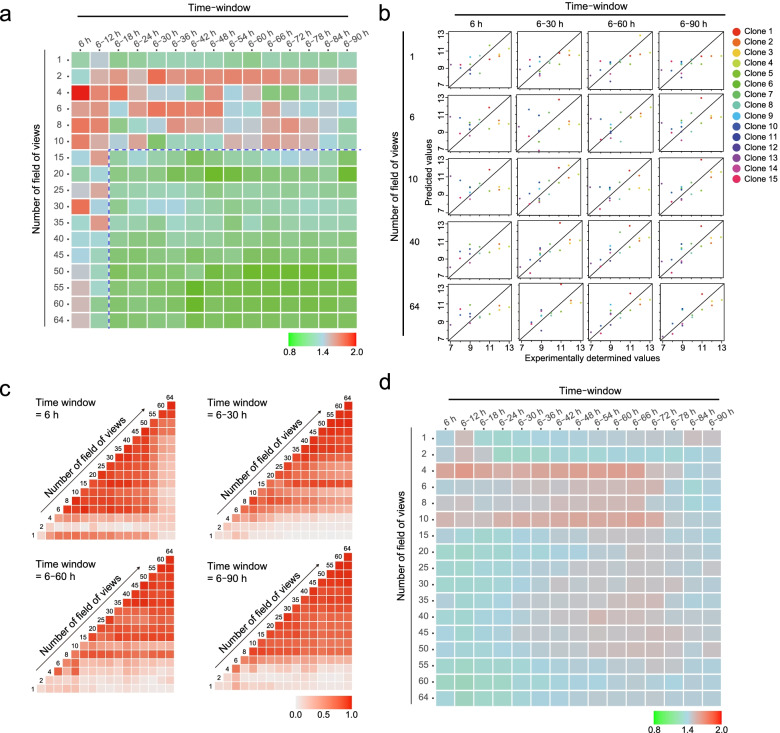
Exhaustive evaluation of the data-usage effect and performances of serial-passage prediction models. **a** Evaluation of data-usage effect with LASSO. The row values represent the number of FOVs used, and the column values represent the time-window size used for training data. The heatmap indicates the RMSE. RMSE < 1.0 is considered a good performing model. **b** Scatter plots to visualize the serial-passage prediction model performances; each dot represents one clone. **c** Comparison of model structures between two pairs of constructed models in **a**. Time windows of 6, 6–30, 6–60, and 6–90 h were selected. The heat map indicates the correlation coefficiencies between all weights on all selected morphological descriptors in the model. The correlation coefficiencies become high if the used descriptor combination is similar. **d** Evaluation of the data-usage effect with RF; the row indicates the number of FOVs used, and the column represents the time-window size used for training data. The heatmap indicates the RMSE. RMSE < 1.0 is considered a good performing model

From the exhaustive examination of both the “time-window effect” and the “FOV-number effect” with least absolute shrinkage and selection operator (LASSO), we found that high-performance prediction models (RMSE < 1.0 shown as green colored heatmap in Fig. 4a) can be obtained with several parameter combinations even with the morphological information in P1 stage cells. The data suggest that the prediction performance can be maintained even if the time window and FOV numbers are reduced. Furthermore, it was deduced that the effect of “FOV-numbers” is more important than that of the “time window” because the time window can be shortened without performance degradation when more than 15 FOVs are collected.

A scatter plot is plotted to further understand the model performance for predicting serial-passage potencies for each clone (Fig. 4b). These data clearly show that our prediction models predict “quantitative values of serial-passage limitation numbers.” Many high-performance prediction models have been developed using different sizes of training data (Fig. 4a, b). However, we suspected that all model structures were randomly different by lacking a common structure because LASSO is the algorithm that creates the best descriptor combination for each dataset; similar performance models with a completely different model structure can be obtained. If the model structure is different in each condition of the data, such modeling result is not robust, and therefore not practical. Thus, we compared all model structure correlations (Fig. 4c) and confirmed that the top-performing models robustly share similar model structures. This result suggested that the relation between the morphological descriptor combination and serial-passage potencies can be modeled with a certain universal combination of morphological descriptors.

A comparison between the highly contributing descriptors shared between different models, i.e., “Correlation_SD (18 h),” “Correlation_SD (6 h),” and “Energy_SD (18 h),” contributed positively to predict the long serial-passage potency clones; “Correlation_mean (6 h),” “Length_SD (18 h),” and “Compactness_SD (18 h)” contributed negatively to predict the short serial-passage potency clones (Supplementary Table 3). Both “correlation” and “energy” are texture descriptors, and therefore, they commonly reflect the three-dimensional pattern and complexity of cells because it changes the intensity profile under phase-contrast microscopy. In practice, the intensity profiles change drastically when cells change their three-dimensional roundness during cell division. Thus, an increase in the “SD of texture” indicates that there are more proliferating cell populations. Thus, the population becomes more homogeneous in texture when the “mean of texture” has a greater effect; this implies that there is a decrease in the number of cellular events that change texture. Length and compactness are shape-related descriptors; therefore, they commonly reflect two-dimensional responses such as elongation and expansion. If such a shape changes with few textural changes, it indicates that the cells use their activity for elongation more instead of for proliferation. Thus, such model structure-derived information suggests that the acquired serial-passage prediction models are not only useful for early detection of their future potency, but are also informative for the quantitative extraction of the morphological rule, which is descriptive and recordable. Thus, such a descriptive understanding of morphological profiles helps escape from the old habits of grasping morphological changes by feeling.

The model did not show a better performance than LASSO in any data usage conditions when we examined the same condition matrix with the nonlinear machine learning model RF (Fig. 4d). These data reflect that the serial-passage potency and morphological information are linearly related.

**Fig. 5:**
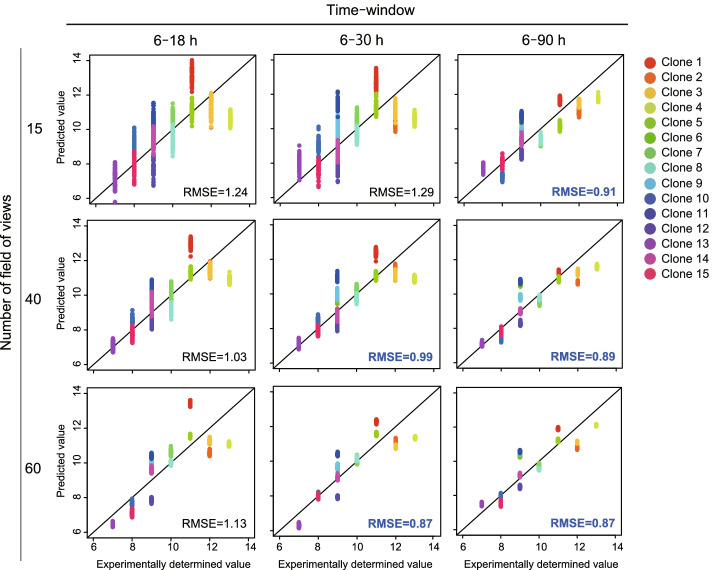
Evaluation of the robustness of serial-passage prediction models against data variation included by the bootstrap FOV selection (50 repeats) and its data-usage effect

Finally, we attempted to confirm the predictive performance of LASSO models in more detail because the model training was based on their raw sample data, which are relatively small in size compared to other fields’ machine learning applications, although we examined two parameter combinations (time window and FOV number). We introduced bootstrap to increase the variations of image-derived morphological profiles for evaluating the extent to which the prediction model can stand robustly to the effect from the data variation. We examine the performance of the prediction models by introducing 50 bootstraps to collect different FOV combinations from the 64-tiling images per sample (Fig. 5). The result indicates that such image-sampling bias introduced by bootstrapping caused performance degradation in some prediction models. However, although the range of the “time-window effect” and “FOV-number effect” for achieving high-performance models (RMSE < 1.0) was narrowed by a more robust model compared to that in Fig. 4a–c, we can minimize the data collection size to 6–30 h with 40 FOVs for the earliest prediction, and 6–90 h with 15 FOVs for the minimum effort prediction while keeping the prediction accuracy (RMSE < 1.0). The FOV number clearly improved the morphological profile robustness when we evaluated such a bootstrap effect on PCA; the accumulation of the morphological profile by a longer time window improved to discriminate between clone differences. Such data-size effect investigations will contribute to designing a more effective process of introducing an image-based quality check in cell bank establishment.

## Discussions

RECs are clonal MSCs selected from human BMMCs, which not only retain the superior qualities of conventionally processed MSCs, but also have characteristics that are advantageous for the practical cell manufacturing process for therapeutic products. In particular, the high proliferative potency of RECs provides a major advantage in developing efficient manufacturing processes for cell therapeutic products. Therefore, in this study, we investigated the possibility of predicting continuous passaging capacity from initial morphological information alone and its most practical construction method so that the capacity of RECs can be evaluated from the initial stage of cell bank construction.

A long unsolved problem in any type of cell culture is determining “the best timing to make cryo-stocks” in the expansion culture. Since most normal cells lose their proliferation potency when cultured in vitro [38, 39], one can only bet on which passage number to end with while making cryostocks. For establishing industrial cell banks, such a betting factor amounts to a significant risk: the low-success-rate expansion culture will incur significant expenses if the cell does not proliferate as expected, and if the cells are cryopreserved too early, the stock will not profit production efficacy. In practice, the bottleneck in the practical cell bank establishment is the effort of recruiting precious donors, and not the effort of making greedy selection of candidate cell banks. However, with RECs, we expect a stricter and more selective process for finalizing the candidate cell bank as a “master cell bank.” Our investigation presents a new concept of using morphological noninvasive analysis as an “in-process analysis tool” for enhancing and optimizing the cell bank establishment process. This concept will help set the best cryostock production timing by balancing “the yield of cells” and “the remaining potency of banked cells” and predicting the future serial-passage potency. Such an approach will help discard the present cell bank design concept, which restricts the passage number using data-less logic.

Although we investigated a method to predict the “serial-passage potency” of RECs, but the continuous passage potency in cMSCs may require some discussion. It is understood that for the induced pluripotent stem cells (iPSCs), the uncontrollable proliferation potency in iPSCs has a negative effect on clinical treatment, for example, the risk of teratoma formation [40, 41]. However, with MSCs, which are known to exhibit limited proliferation potency, their serial-passage potencies are considered with several aspects. If the “candidate cell bank” of this study is prepared as a master cell bank for the creation of further working cell banks, its serial-passage potency would be beneficial to the entire process. However, the serial-passage potency effect should be carefully examined if it is prepared as the final cell bank for implantation. If it profits the efficacy of the final product, it can be an advantage; however, if it negatively affects the efficacy, it will be a risk. In any case, such future potency prediction from the earliest stage of the process will help optimize the final cell bank quality because it can only be evaluated by excessive continuous evaluations for futile REC candidates. Since REC is currently on the path to clinical trials, our next challenge is to validate the effectiveness of such potency predictions and efficiently move forward with product manufacturing.

Finally, our morphology-based future potency prediction on RECs triggers the discussion of whether this developed model can be applied to other MSC cell-bank establishment studies. Currently, we consider that our model is still limited to predicting and evaluating RECs. This interpretation is not attributed to the difference in potencies in RECs and bulk MSCs because we clearly found that the morphological distribution of RECs differs from that of bulk MSCs. Our image-based detailed morphology measurement indicated that the major population of RECs comprises nearly twofold smaller cells compared to the bulk MSCs. Such large size differences would make the prediction model structure, the combination of morphological descriptors, fit for RECs because we use “mean and SD” for reflecting the cell population distribution for our morphological profiles. Furthermore, it is practically difficult for bulk MSCs to conduct “serial-passage tests” for as long as RECs. Such large differences between the native potencies of RECs and bulk MSCs can result in unexpected data bias that can unexpectedly develop serial-passage prediction models that discriminate “RECs or bulk MSCs” from biased data. Therefore, it is a future challenge to investigate such universal morphological characteristics that can be reflected in other stem cells.

## Conclusions

Although our findings are based on a limited number of clones, our investigation of image-based machine learning models was found to introduce a new concept of data-driven process management for a more effective cell bank establishment. Our next challenge will be to expand our morphology-based early cell potency predictions to obtain clones with higher differentiation potencies which closely relate to the therapeutic effects.

## Supplementary Information

**Additional file 1:** **Supplementary Fig. 1.** Schematic pipeline of image processing used in this study.

**Additional file 2:** **Supplementary Table 1.** Basic morphological parameters measured per cell.

**Additional file 3:** **Supplementary Fig. 2.** Growth profiles of conventionally processed bulk MSCs (nine lots).

**Additional file 4:** **Supplementary Table 2**. Morphological descriptors highly contributing to PCA.

**Additional file 5: Supplementary Table 3.** Morphological descriptors used in LASSO models.

## Data Availability

Datasets used and/or analyzed during the current study are available from the corresponding author upon reasonable request.

## Abbreviation

MSC: Mesenchymal stem cell
BMMC: Bone marrow mononuclear cell
REC: Rapidly expanding MSC clone
P1: Passage number 1
P2: Passage number 2
P3: Passage number 3
SD: Standard deviation
PCA: Principal component analysis
PC1: Principal component 1
PC2: Principal component 2
LASSO: Least absolute shrinkage and selection operator
RF: Random Forest
FOV: Field-of-view
RMSE: Root-mean-squared error
iPSC: Induced pluripotent stem cell
