# Supplementary figures and images for: Morphology-based noninvasive early prediction of serial-passage potency enhances the selection of clone-derived high-potency cell bank from mesenchymal stem cells
Source: Inflamm Regen. 2022 Oct 2;42:30. doi: 10.1186/s41232-022-00214-w (PMC9526913; doi:10.1186/s41232-022-00214-w)

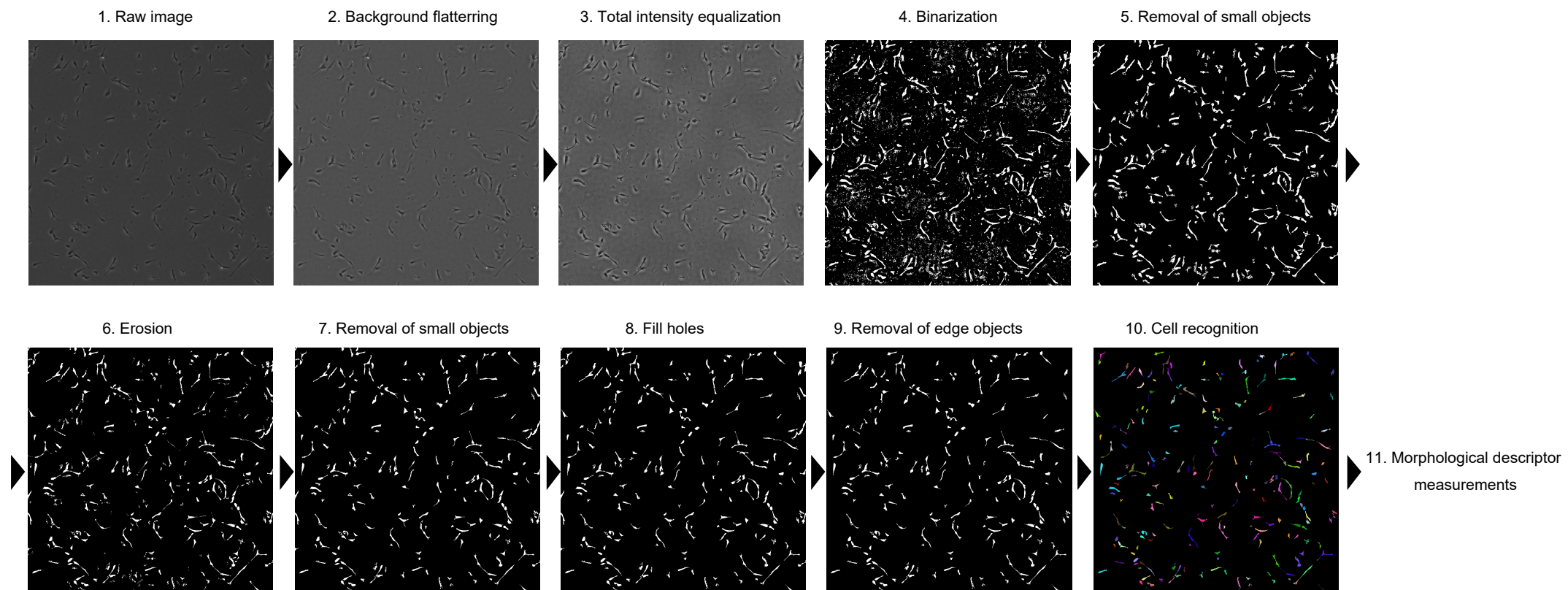

Supplementary Fig. 1

Supplement: Supplementary file 1 — Additional file 1: Supplementary Fig. 1. Schematic pipeline of image processing used in this study. [file 41232_2022_214_MOESM1_ESM.pdf]

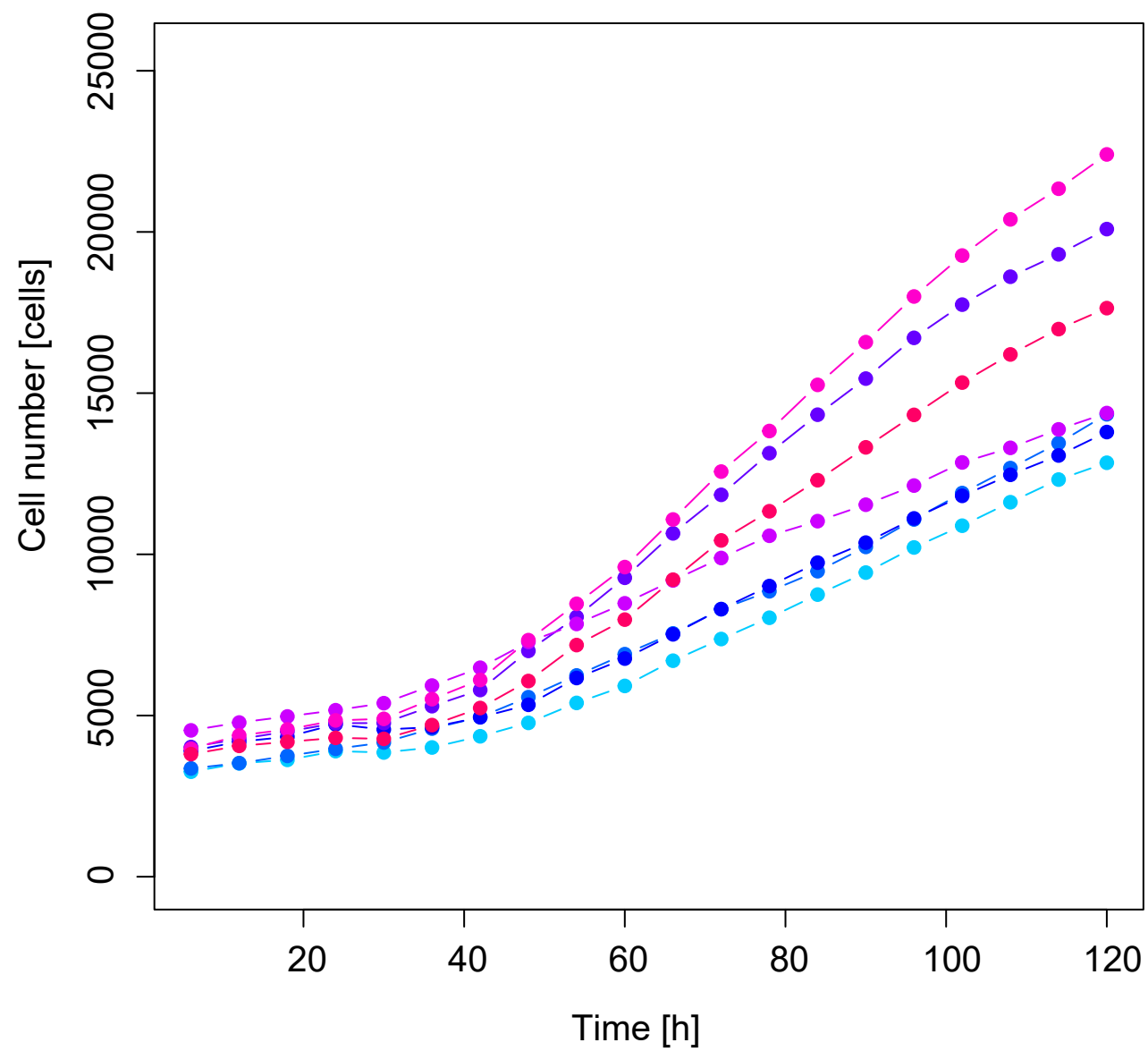

Supplementary Fig. 2 Growth profile of bulk MSCs.

Supplement: Supplementary file 3 — Additional file 3: Supplementary Fig. 2. Growth profiles of conventionally processed bulk MSCs (nine lots). [file 41232_2022_214_MOESM3_ESM.pdf]
