# Supplementary material for: Morphology-based noninvasive early prediction of serial-passage potency enhances the selection of clone-derived high-potency cell bank from mesenchymal stem cells
Source: Inflamm Regen. 2022 Oct 2;42:30. doi: 10.1186/s41232-022-00214-w (PMC9526913; doi:10.1186/s41232-022-00214-w)
Supplement: Supplementary file 2 — Additional file 2: Supplementary Table 1. Basic morphological parameters measured per cell. [file 41232_2022_214_MOESM2_ESM.pdf]

Supplementary Table 1. Basic morphological parameters measured per single cells.

Number	Parameter type	Name	
1	Shape descriptors	Area	Total pixels in the recognized cell region.
2		Compactness	(Perimeter)^2/Area
3		Inner radius	Radius of inscribed circle from the centroid of cell area.
4		Length width ratio	Length/Width
5		Perimeter	The arc length of recognized cell region.
6		Shape factor	4π(Area)/(Perimeter)^2
7	Texture descriptors	Correlation	Gray-Level Co-occurrence Matrix (GLCM) of cell region. $\mathrm{GLCM} = \mathrm{M}$ $\mathrm{Correlation} = \sum_{i,j} \frac{(i - \mu_x)(j - \mu_y)M_i}{\sigma_x \sigma_y}$ $\mu_x = \sum_{i,j} j M_{i,j}$ $\mu_y = \sum_{i,j} i M_{i,j}$
8		Energy	Gray-Level Co-occurrence Matrix (GLCM) of cell region. $\mathrm{GLCM} = \mathrm{M}$ $\mathrm{Energy} = \sum_{i,j} M_{i,j}^2$
9		Entropy	Gray-Level Co-occurrence Matrix (GLCM) of cell region. $\mathrm{GLCM} = \mathrm{M}$ $\mathrm{Entropy} = -\sum_{i,j} M_{ij} \log M_{i,j}$
10		Inertia	Gray-Level Co-occurrence Matrix (GLCM) of cell region.

			$GLCM = M$ $Inertia = \sum_{i,j}(i - j)^2 M_{ij}$
11		Homogony	Gray-Level Co-occurrence Matrix (GLCM) of cell region. $GLCM = M$ $Homogony = \sum_{i,j} \frac{M_{i,j}}{1 + \lvert i - j \rvert}$
12		Intensity SD	Standard deviation of intensities of pixels in cell region.
