# Supplementary material for: Morphology-based noninvasive early prediction of serial-passage potency enhances the selection of clone-derived high-potency cell bank from mesenchymal stem cells
Source: Inflamm Regen. 2022 Oct 2;42:30. doi: 10.1186/s41232-022-00214-w (PMC9526913; doi:10.1186/s41232-022-00214-w)
Supplement: Supplementary file 4 — Additional file 4: Supplementary Table 2. Morphological descriptors highly contributing to PCA. [file 41232_2022_214_MOESM4_ESM.pdf]

Supplementary Table 2. Morphological descriptors highly contributing to PCA.

PC1		PC2	
Parameters	Factor loadings	Parameters	Factor loadings
84h_mean_energy	0.08	24h_mean_length_width_ratio	0.11
78h_mean_energy	0.08	30h_mean_length_width_ratio	0.10
72h_mean_energy	0.08	18h_mean_perimeter	-0.10
54h_mean_length	-0.08	12h_mean_length	-0.10
60h_mean_perimeter	-0.08	18h_sd_width	-0.10
78h_mean_intensity_sd	-0.08	24h_sd_perimeter	-0.10
60h_mean_length	-0.08	24h_sd_length	-0.10
54h_mean_area	-0.08	18h_sd_compactness	-0.11
72h_mean_intensity_sd	-0.08	18h_mean_length	-0.11
54h_mean_perimeter	-0.08	24h_mean_length	-0.11
