# Supplementary material for: Morphology-based noninvasive early prediction of serial-passage potency enhances the selection of clone-derived high-potency cell bank from mesenchymal stem cells
Source: Inflamm Regen. 2022 Oct 2;42:30. doi: 10.1186/s41232-022-00214-w (PMC9526913; doi:10.1186/s41232-022-00214-w)
Supplement: Supplementary file 5 — Additional file 5: Supplementary Table 3. Morphological descriptors used in LASSO models. [file 41232_2022_214_MOESM5_ESM.pdf]

Supplementary Table 3. Morphological descriptors used in LASSO models.

Model using 15FOVs_6-18h		Model using 40FOVs_6-18h		Model using 60FOVs_6-18h		Model using 60FOVs_6-90h	
Parameter	Coefficient	Parameter	Coefficient	Parameter	Coefficient	Parameter	Coefficient
18h_mean_perimeter	1.781	18h_mean_energy	1.191	18h_mean_area	**0.753**	36h_mean_intensity_total	0.226
6h_mean_area	1.391	18h_mean_intensity_sd	0.865	**6h_sd_correlation***	**0.582**	18h_sd_correlation	0.075
18h_mean_intensity_sd	1.104	18h_sd_perimeter	0.845	**18h_sd_energy****	**0.559**	**6h_sd_correlation***	**0.069**
18h_mean_energy	1.092	18h_mean_area	0.668	18h_mean_energy	0.436	12h_sd_correlation	0.057
6h_mean_length	0.904	**6h_sd_correlation***	**0.629**	18h_sd_width	0.391	36h_mean_width	0.046
**6h_sd_correlation***	**0.662**	6h_mean_area	0.447	18h_mean_intensity_sd	0.345	48h_mean_intensity_total	0.041
18h_sd_perimeter	0.587	**18h_sd_energy****	**0.364**	12h_mean_area	0.261	24h_mean_width	0.040
6h_mean_width	0.537	6h_sd_energy	0.325	6h_mean_area	0.156	**18h_sd_energy****	**0.039**
18h_mean_area	0.522	12h_mean_width	0.319	6h_mean_compactness	0.136	30h_mean_width	0.036
6h_sd_intensity_total	0.485	12h_sd_perimeter	0.295	6h_mean_length	0.127	78h_sd_intensity_mean	0.028
12h_mean_length_width_ratio	-0.539	18h_mean_homogeneity	-0.437	18h_mean_homogeneity	-0.220	**6h_mean_correlation****	**-0.083**
6h_mean_energy	-0.565	6h_mean_perimeter	-0.448	6h_sd_perimeter	**-0.231**	54h_sd_length_width_ratio	-0.097
**18h_mean_compactness***	**-0.642**	**6h_mean_correlation****	**-0.479**	18h_mean_intensity_total	-0.269	42h_mean_length_width_ratio	-0.109
18h_sd_length	-0.673	6h_mean_length_width_ratio	-0.493	12h_mean_correlation	-0.287	90h_mean_area	-0.110
12h_mean_homogeneity	-0.683	18h_sd_intensity_sd	-0.498	6h_mean_length_width_ratio	-0.412	**18h_sd_length***	**-0.112**
12h_mean_perimeter	-0.784	12h_mean_perimeter	-0.519	12h_sd_length	-0.431	48h_mean_length_width_ratio	-0.132
18h_mean_length_width_ratio	-0.865	12h_mean_length_width_ratio	-0.577	**6h_mean_correlation****	**-0.484**	**18h_sd_compactness***	**-0.242**
6h_mean_perimeter	-1.135	**18h_sd_compactness***	**-0.688**	18h_mean_length_width_ratio	**-0.511**	24h_sd_compactness	-0.265
6h_mean_intensity_total	-1.594	18h_mean_length_width_ratio	-0.825	**18h_sd_compactness***	**-0.614**	60h_mean_length_width_ratio	-0.276
**18h_mean_length***	**-1.757**	**18h_sd_length***	**-0.917**	**18h_sd_length***	**-0.701**	24h_sd_length	-0.440

*Blue: Commonly used effective descriptor shared in models using small to large data-size.
**Red: Commonly used effective descriptor shared in models using larger data-size.
